# Inflammatory Cytokines: Potential Biomarkers of Immunologic Dysfunction in Autism Spectrum Disorders

**DOI:** 10.1155/2015/531518

**Published:** 2015-02-01

**Authors:** Ningan Xu, Xiaohong Li, Yan Zhong

**Affiliations:** ^1^Department of Children's Health, Hunan Children's Hospital, Hunan, China; ^2^Department of Neurochemistry, NY State Institute for Basic Research in Developmental Disabilities, New York, NY 10314, USA

## Abstract

Autism is a disorder of neurobiological origin characterized by problems in communication and social skills and repetitive behavior. After more than six decades of research, the etiology of autism remains unknown, and no biomarkers have been proven to be characteristic of autism. A number of studies have shown that the cytokine levels in the blood, brain, and cerebrospinal fluid (CSF) of autistic subjects differ from that of healthy individuals; for example, a series of studies suggests that interleukin-6 (IL-6), tumor necrosis factor-*α* (TNF-*α*), and interferon-*γ* (IFN-*γ*) are significantly elevated in different tissues in autistic subjects. However, the expression of some cytokines, such as IL-1, IL-2, transforming growth factor-*β* (TGF-*β*), and granulocyte-macrophage colony-stimulating factor (GM-CSF), is controversial, and different studies have found various results in different tissues. In this review, we focused on several types of proinflammatory and anti-inflammatory cytokines that might affect different cell signal pathways and play a role in the pathophysiological mechanism of autistic spectrum disorders.

## 1. Introduction

Autism spectrum disorders (ASD) are a complex group of severe neurodevelopmental disorders that affect over 1% of children in the United States [1]. Typical symptoms of autism include impairments in social interaction, deficits in verbal and nonverbal communication, and repetitive behaviors and restricted interests [2]. Although the exact etiology of the disorder has not been identified, a link between altered immune responses and ASD was first recognized nearly 40 years ago. Neurobiological studies in ASD have highlighted pathways involved in neural development, synapse plasticity, structural brain abnormalities, cognition, and behavior. In addition, abnormalities in the cellular immune response have also been reported in children with autism; in particular, reduced cytotoxic activity and elevated levels of selected proinflammatory cytokines produced by peripheral blood mononuclear cells, such as tumor necrosis factor (TNF-*α*) and IL1*β*, have been shown to disrupt neurodevelopment [3, 4].

Cytokines are polypeptides that are 8–25 kDa in size and include interleukins, chemokines, interferons, tumor necrosis factors (TNFs), and growth factors. Cytokines have been shown to regulate the growth and cell proliferation of neuronal tissue and to modulate host responses to infection, injury, inflammation, and diseases of uncertain etiology [5]. The dynamic expression of different cytokines can modify immune system function; for example, increased levels of interferon-gamma (IFN-*γ*) and IL-12 can induce inflammation, whereas increased production of TGF-*β* can negatively regulate inflammation [6]. A number of recent studies have demonstrated that the levels of various inflammatory cytokines differ in the blood mononuclear cells, serum, plasma, brain tissue, and cerebrospinal fluid of autistic subjects compared with normal subjects, which might impair immune capacity in the central nervous system (CNS) and stimulate the production and activation of microglia in the brain [7, 8]. Microglia are the unique resident immune cells of the CNS; they act as primary mediators of inflammation, participating in immune surveillance of the CNS and synaptic pruning during normal neurodevelopment. Mounting evidence indicates that chronic microglial activation might also contribute to the development and progression of neurodegenerative disorders. Active microglia can induce the production of pro-inflammatory cytokines such as IL-1, IL-6, and TNF-*α*, which are typically intended to prevent further damage to brain tissue. However, abnormally activated microglia can sometimes be toxic to neurons and other glial cells. Microglia cell activation is a prominent feature of autism, and there is a complex interaction between microglial cells and cytokines. Wei et al. found that IL-6 elevation can modulate autism-like behaviors through impairments of synapse formation, dendritic spine development, and neuronal circuit balance [7].

In this review, we focused on several types of proinflammatory and anti-inflammatory cytokines that might affect different cell signal pathways and play a role in the pathogenic mechanism that might be responsible for autism (Table 1).

## 2. Cytokines and Autism

### 2.1. Interleukins

#### 2.1.1. Interleukin-1

Interleukin-1 (IL-1) consists of two different proteins, IL-1*α* and IL-1*β*, which exert their proinflammatory effects by binding to type-I IL-1 receptors (IL-1 RI), whereas the type II IL-1 receptor (IL-1RII) is thought to act as a decoy receptor that does not participate in active IL-1 signaling [9, 10]. IL-1*β* is produced, processed, and secreted from activated immune cells and plays a major role in the initiation of local and systemic inflammatory processes. Signaling mediated by IL-1RI can activate the myeloid differentiation response gene 88 (MyD88) and tumor necrosis factor-associated factor 6 (TRAF6), leading to the activation of a number of different kinases, including IL-1 receptor associated kinases 1 and 4 (IRAK1, IRAK4), transforming growth factor *β*-activated kinase (TAK), and members of the mitogen activated protein kinase (MAPK) cascade, including p38, c-Jun N-terminal kinases (JNK), and extracellular signal-regulated kinases 1/2 (ERK1/2) [11].

In 1991, Singh et al. first measured the serum levels of soluble IL-1 and found that the level did not differ between the subjects with autism and the control subjects [12]. Croonenberghs et al. (2002) demonstrated that there is also no difference in the IL-1 receptor between autism and the control subjects [13]. However, in vitro, Jyonouchi and his group observed that stimulated peripheral blood mononuclear cells (PBMCs) from children with autism secrete significantly higher amounts of soluble IL-1*β* compared with normal controls [14]. In an animal model, maternal exposure to poly-(I:C) or lipopolysaccharide (LPS) can induce a significant increase of IL-1*β* in amniotic fluid, placentas, and fetal brains. IL-1*β* has also been shown to play a key role in mediating severe placental damage and neurodevelopmental anomalies in offspring [15, 16]. IL-1*β* showed the highest concentration levels in fetal brains and was the only cytokine that was significantly upregulated 24 h after maternal poly (I:C) injection, suggesting that IL-1*β* may have a deleterious impact on central nervous system development. However, these studies did not evaluate the social behavior of offspring, so further study is needed to determine whether there is a link between the development of autism and elevated IL-1*β* levels.

X-linked interleukin-1 receptor accessory protein like-1 (IL1RAPL1) is a member of the interleukin-1 receptor family and is similar to the interleukin-1 accessory proteins. Piton et al. (2008) first reported the function of the resulting truncated IL1RAPL1 protein, which is severely altered in hippocampal neurons, by measuring its effect on neurite outgrowth activity [17]. The mutations in IL1RAPL1 cause a spectrum of neurological impairments ranging from mental retardation to high-functioning autism. Pavlowsky and colleagues also reported a novel partner of IL1RAPL1, PSD-95, a major scaffold protein of excitatory synapses, and showed that IL1RAPL1 regulates the dendritic spine number and PSD-95 localization to synapses [18]. Further investigation showed that IL-1-induced activation of the JNK pathway in neurons is mediated by IL1RAPL1. This finding indicates that a novel pathophysiological mechanism underlying cognitive impairment could be associated with alterations of the JNK pathway in response to IL-1 and hence lead to the mislocalization of PSD-95, which subsequently result in abnormal synaptic organization and function [19].

#### 2.1.2. Interleukin-2

Interleukin-2 (IL-2) is a well-known cytokine that plays an important role in multiple immunoregulatory functions related to T-cells in peripheral and CNS [20, 21]. The IL-2 receptor consists of three subunits, IL-2R*α*, *β* (IL-2R*β*), and *γ* (IL-2R*γ*), which can deliver biochemical signals to the cell interior. They affect different signal pathways, such as the MAPK pathway, the phosphoinositide 3-kinase (PI3K) pathway, and the JAK-STAT pathway [22]. IL-2 in the brain is potentially produced by neurons and astrocytes and is widely distributed throughout the brain along with its receptors. There is a large body of literature indicating that IL-2 may be involved in CNS development, normal brain physiology, and homeostatic repair mechanisms as well as brain dysfunction and neurodegenerative processes. For instance, it has been shown that prolonged exposure to IL-2 in cancer patients can result in cognitive dysfunction, altered behavior, and other negative neuropsychiatric side effects [23]. In addition, IL-2 levels have been found to be elevated in the postmortem brains of patients with Alzheimer's disease compared with normal control subjects [24].

Singh et al. found that serum concentrations of soluble IL-2 were significantly higher in autistic children compared with normal controls. In addition, they showed that stimulated peripheral blood mononuclear cells (PBMCs) from children with autism secrete significantly higher amounts of IL-2, whereas soluble IL-2 receptor levels did not differ between autistic and control subjects [12]. However, more recent studies have reported that IL-2 levels did not differ significantly between subjects with autism and control groups [25, 26]. Vojdani and colleagues measured NK cell activity in 1027 blood samples from autistic children obtained from ten clinics and compared the results to those of 113 healthy controls. The authors found that 45% of the autistic children exhibited low NK cell activity. In addition, they cultured lymphocytes from autistic children with low or high NK cell activity and with or without IL-2 and determined that the induction of NK cell activity by IL-2 was more pronounced in a subgroup with very low NK cell activity [27].

#### 2.1.3. IL-6

IL-6 is characterized as a 26 kDa glycoprotein that can trigger cellular responses that mediate inflammation, neurogenesis, gliogenesis, cell growth, cell survival, myelination, and demyelination in the CNS [28, 29]. The IL-6 receptor protein complex consists of one nonsignaling, membrane-associated *α* subunit (IL-6R) and two gp130 subunits responsible for signal transduction [30, 31]. Ligand binding of this protein complex results in the homodimerization of gp130 and activates multiple signaling mechanisms, including JAK/STAT, PI3K/Akt, and the Ras/Raf/MAPK pathways [31–34].

Recently, the role of maternal immune activation (MIA) has become a hot topic in autism research. Several groups have found that the developing fetus's exposure to maternal cytokines precipitates neurological, immunological, and behavioral abnormalities in the offspring [35, 36]. These effects require a key mediator, IL-6. Maternal injection with IL-6 alone is sufficient to cause abnormal behavior in the offspring following maternal poly-(I:C) injection or respiratory infection. Conversely, IL-6 inhibition was sufficient to attenuate the behavioral deficits caused by MIA [35, 37]. In our laboratory, we found that mice with elevated IL-6 in the brain display many autistic features, including impaired cognitive abilities, learning deficits, abnormal anxiety traits, and habituations and decreased social interactions. We also examined the development of synapses and found that IL-6 elevation altered excitatory and inhibitory synaptic formations, disrupted the balance of excitatory/inhibitory synaptic transmissions, and resulted in an abnormal change in the shape, length and distributing pattern of dendritic spines. These findings suggest that IL-6 elevation in the brain could mediate autistic-like behaviors, possibly through the disruption of the neural circuitry balance and impairments of synaptic plasticity [7]. Hsiao and Patterson confirmed that MIA elevates IL-6 protein and mRNA expression in the placenta and found that maternally derived IL-6 mediates JAK/STAT3 pathway activation specifically in the spongiotrophoblast layer, a fetal compartment of the placenta, which results in the expression of acute-phase genes [38]. Furthermore, Hsiao and Patterson also demonstrated an IL-6-dependent dysregulation of the growth hormone/insulin-like growth factor (GH-IGF) axis in MIA placentas that was characterized by decreased levels of GH and IGF1 mRNA with corresponding decreases in placental IGF1 and IGF-binding protein 3 (IGFBP3).

Singh first examined the plasma levels of IL-6 and found no significant differences between subjects with autism and controls [39]. However, Vargas' group demonstrated that IL-6 was increased in autistic brains [8]. In our recent studies, we also found that IL-6 was significantly increased in the cerebella of autistic subjects [40]. The cerebellum was suggested as a main focus of neuroinflammation in autism, and the selective vulnerability of the Purkinje cells may play a role in the etiopathogenesis of autism [41]. In further studies, we investigated how IL-6 affects neural cell development and function by transfecting cultured mouse cerebellar granule cells with an IL-6 viral expression vector. We demonstrated that IL-6 overexpression in granule cells caused impairments in granule cell adhesion and migration but had little effect on the formation of dendritic spines or granule cell apoptosis [40].

**Figure 1:**
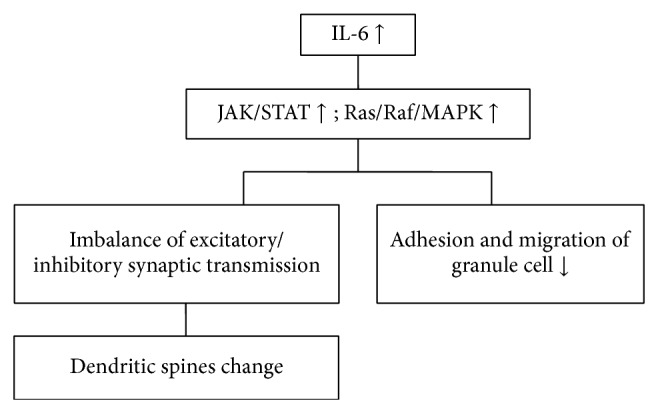
The potential role of IL-6 in the pathogenesis of ASD.

These findings suggest that IL-6 not only plays an important role in the etiology of autism, but may provide a potential biological marker that enables the early diagnosis of the disorder and earlier therapeutic intervention (Figure 1). Because IL-6 can attenuate abnormal behavior caused by MIA, it might be a potential therapy target in the future.

### 2.2. Chemokines

The chemokines are a family of small cytokines that play an important role in the development of lymphocytes, including their recruitment and trafficking to specific tissue compartments. In addition to acting as key chemotactic factors in the immune system, chemokines and their receptors are critical for dictating the movement of leukocytes into the CNS in both healthy and diseased individuals [42]. Chemokines have been shown to regulate neuronal cell migration, proliferation, and neuronal cell differentiation and are involved in the communication between neurons and microglia [43, 44].

*Monocyte chemoattractant protein 1* (MCP-1/CCL2) is known to mediate monocyte and T-cell activation and trafficking into areas of tissue injury [45]. In the CNS, MCP-1 can modulate the recruitment of myeloid cells to injury or inflammation sites, and it is increased in brain in instances of ischemia, Alzheimer's disease, and experimental autoimmune encephalomyelitis [42]. Different groups have measured MCP-1 levels in the brain, CSF, and plasma of individuals with autism and found that MCP-1 was elevated in individuals with autism. Elevated MCP-1 production was associated with higher aberrant behavior scores and more impaired developmental and adaptive function [8, 42]. Abdallah and colleagues examined the chemokine levels in amniotic fluid (AF) samples from individuals diagnosed with autism and controls [46]. The result showed an increased risk for autism with elevated MCP-1 compared with controls. In an MIA model, MCP-1 was significantly upregulated after the injection of poly-(I:C). MCP-1 can enhance neuronal excitability and synaptic transmission in hippocampal neurons and can modulate neuronal physiology [47, 48]. Taken together, these findings suggest that elevated MCP-1 in the brain might be linked to microglial activation and to the recruitment of monocytes/macrophages to areas of neurodegeneration.

*Osteopontin* (OPN) is a 60 kDa phosphoprotein that plays different roles, including initiating inflammation, cell adhesion, chemotaxis, immune responses, and protection against apoptosis, depending on its intracellular or extracellular localization [49]. OPN is also involved in regulating chronic inflammatory and autoimmune diseases, including multiple sclerosis (MS), rheumatoid arthritis, inflammatory bowel disease, systemic lupus erythematosus, and type I diabetes [50–53]. Recently, studies have demonstrated that OPN also plays a role in the development of neurodegenerative diseases, such as Parkinson's and Alzheimer's disease. However, OPN can trigger neuronal toxicity and death in some contexts and can function as a neuroprotectant in others [54]. AL-Ayadhi and Mostafa (2011) measured serum OPN levels using ELISA in 42 autistic children and 42 matched healthy children [55]. The authors found that the autistic children had significantly higher serum OPN levels compared with the healthy controls, and the autistic children's serum OPN levels had significant positive correlations with their Childhood Autism Rating Scale (CARS) scores.

However, it is difficult to determine the exact roles of specific chemokines during neurodevelopment because of the high degree of interaction between different chemokines and their receptors. At present, the pathologic mechanism of chemokines in children with autism is not clear. However, a number of studies have reported abnormal chemokine production in the brain, CSF, and/or plasma of autistic individuals, suggesting that chemokines might be involved in aberrant neuronal development that could lead to altered early brain development and function.

### 2.3. TNF-*α*

Tumor necrosis factor-*α* (TNF-*α*) is a cytokine involved in systemic inflammation. It is a member of a group of cytokines that stimulate the acute phase reaction. TNF-*α* is produced mainly by activated macrophages (M1), although it can be produced by other cell types, such as CD4+ lymphocytes and NK cells. TNF can bind two receptors, TNF receptor type 1 (TNF-R1) and TNF receptor type 2 (TNF-R2). Binding with ligand, TNF-*α* can activate NF-*κ*B, MAPK, and the apoptosis signaling pathway [56].

In 1996, Singh demonstrated that plasma TNF-*α* did not significantly differ between autistic subjects and normal controls [39]. However, Jyonouchi and collaborators tested 71 autistic children aged 2–14 years and compared them with healthy siblings and other controls [14]. The authors found that TNF-*α* was elevated in the autistic subjects. Their study showed that PBMCs activated by LPS produced higher levels of TNF-*α*, IL-1*β*, and/or IL-6 in most autistic children (83.1%) compared with the control group. The investigators concluded that a majority of the autistic children in the group, especially those with increased TNF-*α*, exhibited excessive or poorly regulated innate immune responses. In addition, Chez et al. (2007) detected elevated TNF-*α* in the cerebrospinal fluid of autistic children [4]. In 2009, our lab found that TNF-*α* was significantly increased in the brains of autistic subjects [26]. Furthermore, Ashwood and colleagues used polyhydroxyalkanoates (PHA) and tetanus to stimulate PBMCs from autistic subjects and controls to compare group-associated cellular responses. They found that TNF-*α* production was significantly increased in the autistic subjects [57].

TNF-*α* was specifically selected to activate the classical NF-*κ*B signaling cascade of the innate immune system and to stimulate the nuclear translocation of NF-*κ*B in primary cortical neurons [58]. NF-*κ*B DNA binding activity was significantly increased in the peripheral blood samples of children with autism [59]. NF-*κ*B has also been shown to be aberrantly expressed in the orbitofrontal cortex in patients with autism, which indicates that NF-*κ*B could be part of a putative molecular cascade leading to inflammation in brain regions associated with the behavioral and clinical symptoms of autism [60]. However, our laboratory results show that the expression of NF-*κ*B (p65) and the phosphorylation/activation of NF-*κ*B (p65) at Ser536 are not significantly changed in the cerebellum and cortex of both autistic subjects and BTBR mice in an autism model [61]. These findings imply that NF-*κ*B may be involved in the abnormal inflammatory response processes suggested in autistic brain but do not play an important part. Our results suggest that TNF-*α* might affect the progress of autism through another pathway, such as the MAPK/JNK pathway.

### 2.4. IFN-*γ*

IFN-*γ* is critical for innate and adaptive immunity against viral and intracellular bacterial infections, and it plays a role in tumor control. Cellular responses to IFN-*γ* are activated through its interaction with a heterodimeric receptor consisting of interferon gamma receptor 1 (IFNGR1) and interferon gamma receptor 2 (IFNGR2). IFN-*γ* binding to the receptor activates the JAK-STAT pathway [62]. Aberrant IFN-*γ* expression is associated with a number of autoinflammatory and autoimmune diseases [63].

In 1996, Singh found significantly elevated IFN-*γ* levels in the plasma of 20 autistic children compared with 20 healthy controls [39]. Subsequently, Croonenberghs and colleagues confirmed the results [13]. Recently, our laboratory also demonstrated that the concentration of IFN-*γ* was significantly increased in the brains of autistic subjects compared with normal control subjects [26]. Another study detected elevated serum IFN-*γ* in women who had given birth to a child who was later diagnosed with autism [64]. However, Sweeten et al. (2004) demonstrated that the plasma levels of IFN-*γ* did not differ between the autistic group and control group, but they did correlate positively with plasma nitric oxide measures in the autistic group [65].

It is known that under normal circumstances, pregnancy shifts the maternal immune system toward a more tolerant stage, causing an overall decrease in proinflammatory cytokine trajectories in the innate and adaptive arms of the immune system and an increase in counter regulatory cytokines [66]. Mothers of children with autism demonstrated increased levels of the inflammatory cytokine IFN-*γ*, which may indicate an atypical immune state during gestation [64]. Furthermore, in addition to maternal abnormality of IFN-*γ*, there is also elevated IFN-*γ* in autistic children. These findings support the hypothesis that IFN-*γ* is involved in MIA and plays a role in the pathologic mechanism that may be responsible for autism.

### 2.5. Growth Factors

#### 2.5.1. Transforming Growth Factor-*β* (TGF-*β*)

TGF-*β*1 is one of the most important immune regulators that can effectively control diverse aspects of the immune response. It plays an important role in autoimmune diseases such as myasthenia gravis-related ophthalmoparesis, autoimmune diabetes, and MS [67–70]. In the CNS, TGF-*β*1 is widely recognized as an injury-related cytokine specifically associated with astrocyte scar formation in response to brain injury. In recent years, emerging data regarding TGF-*β*1 and its signaling molecules have suggested that, in addition to its role in brain injury, TGF-*β*1 might be a crucial regulator of CNS development [71]. Considering the importance of TGF*β*1 in immune response and nervous system development, a number of studies have investigated whether TGF-*β*1 alters in autistic subjects. Vargas et al. found that TGF-*β*1 was significantly increased in certain cell line specimens from autistic subjects [8]. However, decreased TGF-*β*1 levels have been observed in the plasma of autistic individuals [67, 72]. In addition, there were significant correlations between clinical scores and TGF*β*1 levels; lower TGF*β*1 levels were associated with reduced adaptive behaviors and worse behavioral symptoms. These findings suggest that immune responses in autism may be inappropriately regulated because of reductions in TGF*β*1, and such immune dysregulation may predispose individuals to the development of possible autoimmune responses and/or adverse neuroimmune interactions during critical windows in development.

In an animal study, Depino and colleagues found that overexpression of TGF-*β*1 during postnatal can lead to ASD-related behaviors, such as decreased social interaction, increased self-grooming, and depression-related behaviors [73]. However, adult hippocampal levels of murine TGF-*β*1 were reduced in animals that were injected with AdTGF at PD14 compared with Adbgal-injected mice. In addition, the study found that ASD-related behaviors correlate with the long-term downregulation of TGF-*β*1 and IL-6 expression in the dentate gyrus and with decreases in the mRNA levels of the synaptic protein neuroligin 3 and the number of Reelin-positive neurons in the dentate gyrus. Conversely, chronic expression of TGF-*β*1 during adulthood leads to transient opposite effects on abnormal social behaviors: it increases social interaction and decreases repetitive behavior. These results indicate that hippocampal TGF-*β*1 plays an important role in the programming and modulation of social interaction, repetitive behavior, and depression-related behavior.

#### 2.5.2. Epidermal Growth Factor (EGF)

EGF is a growth factor that stimulates cell growth, proliferation, and differentiation by binding to its receptor EGFR. EGF can be detected in all regions of the developing and adult brain and in a majority of the neurons and developing astrocytes [74]. EGF has neurotrophic effects on cultured cortical neurons and stimulates neurite outgrowth in dopaminergic neuron cultures [75]. Transgenic mice lacking the EGF receptor (EGFR) develop neurodegenerative disease and die within the first month of life [76]. Researchers examined the association of EGF, TGF-*β*1, and HGF genes with autism in a trio association study using DNA samples from families recruited to the Autism Genetic Resource Exchange [77]. Their results revealed a significant haplotypic association between EGF and autism, but no significant SNP or haplotypic associations were observed for TGF-*β*1 or HGF. These results suggest a possible role of EGF in the pathogenesis of autism. In 2007, a research group measured serum levels of EGF in 17 male subjects with high-functioning autism and 18 age-matched healthy male subjects [78]. They found that the serum EGF levels in the subjects with high-functioning autism were significantly lower compared with those of the normal control subjects. However, there were no correlations between serum EGF levels and clinical variables in the subjects with autism. By contrast, other researchers found that the serum EGF levels in subjects with autism were significantly higher than those of normal control subjects. There were also no correlations between serum EGF levels and clinical variables in the subjects with autism [79, 80].

#### 2.5.3. Brain-Derived Neurotrophic Factor (BDNF)

BDNF is a member of the nerve growth factor family (neurotrophins); it contributes to prenatal and postnatal brain development [81]. BDNF supports neuronal survival, maintains various neuronal activities, modulates neurotransmitter release, participates in neuronal plasticity, and mediates long-term potentiation and memory fixation [82]. Several studies have examined BDNF in the blood and serum of autistic subjects. Nelson and Miyazaki reported higher BDNF levels in archived samples of neonatal blood and serum obtained from autistic subjects compared with normal controls [83, 84]. However, in a carefully constructed large-sample study, the results demonstrated that the mean levels of BDNF were significantly lower in the serum of 0- to 9-year-old autistic children compared with age-matched healthy controls or teenagers or adults [85]. Our study found that the concentrations of BDNF and Bcl2 were reduced in the frontal cerebral cortex of autistic subjects compared with age-matched controls [86]. We also found that the expression and activation of Akt kinase, which regulates Bcl2, are significantly decreased in the autistic brain. The downregulation of Akt may result from a decreased concentration of BDNF, which is the growth factor that modulates Akt activities. We thus reason that the downregulation of the BDNF-Akt-Bcl2 antiapoptotic signaling pathway in the autistic brain could be one of the underlying mechanisms responsible for the pathogenesis of autism.

#### 2.5.4. GM-CSF

Granulocyte-macrophage colony-stimulating factor (GM-CSF) is a cytokine that regulates the survival, proliferation, differentiation, and functional activity of many myeloid hemopoietic cells; it also regulates dendritic cell and T-cell function [87]. Ashwood and colleagues investigated adaptive cellular immune function in 66 children with a confirmed diagnosis of ASD and 73 confirmed typically developing (TD) controls aged 2 to 5 years [57]. In this study, the in vitro stimulation of PBMC with PHA and tetanus was used to compare group-associated cellular responses. The results showed that GM-CSF, TNF-*α*, and IL-13 production were significantly increased, whereas IL-12p40 was decreased following PHA stimulation in autism relative to TD controls. In addition, the study also found that induced cytokine production was associated with altered behaviors in autistic children: increased proinflammatory or T(h)1 cytokines were associated with greater impairments in the core features of autism and more aberrant behaviors. By contrast, the production of GM-CSF and T(h)2 cytokines was associated with better cognitive and adaptive function. Our laboratory also found that GM-CSF was elevated in the brains of autistic patients compared with controls [26]. However, a study by Manzardo's group showed that the GM-CSF levels were significantly lower in the plasma of children with autism compared with unrelated siblings without autism [88]. GM-CSF can upregulate neuronal receptors on CD34+ hematopoietic stem progenitor cells to enhance responses to neurotransmitters, stimulate neurite growth, and promote axonal regeneration [89–91]. GM-CSF can also promote neuronal differentiation of adult neuronal stem cells and act as a neuronal growth factor in the brain [92]. By contrast, GM-CSF receptors are present on microglia, astrocytes, neurons, and oligodendrocytes. The blockade of GM-CSF receptors with anti-GM-CSF antibodies suppresses microglia activity [93]. These findings suggest that the reduction of microglia activity by GM-CSF receptor blockade may have important anti-inflammatory effects and implications for therapy. Further investigation of the role of GM-CSF in immune responses and neurodevelopment in autism is warranted.

## 3. Conclusion

The studies published to date that have explored possible cytokines abnormalities in autism have been limited by small sample sizes, study design issues, and conflicting results. Although many inconsistencies and controversies exist within these studies, the available data indicate a possible relationship between cytokine alterations and autism. However, systematic investigations of the neuroimmunological factors in autism are needed and will provide a better understanding of the underlying mechanisms responsible for the pathogenesis of autism, thus improving diagnosis and therapy.

## Figures and Tables

**Table 1:** The expression of cytokines in different samples of autistic patients.

Cytokines		Sample	Expression	References
Interleukins	IL-1	Serum	—	Singh et al. (1991) [12]
	IL-1R	Serum	—	Croonenberghs et al. (2002) [13]
	IL-1*β*	PBMC	↑	Jyonouchi et al. (2001) [14]
	IL-2	Serum; PBMC	↑	Singh et al. (1991) [12]
	IL-2R	PBMC	—	Singh et al. (1991) [12]
	IL-2	Plasma	—	Ashwood et al. (2010) [25]
	IL-2	Brain	—	Li et al. (2009) [26]
	IL-6	Plasma	—	Singh (1996) [39]
	IL-6	Brain	↑	Vargas et al. (2005) [8] Wei et al. (2011) [40]

Chemokines	MCP-1/CCL2	Brain; CSF plasma	↑	Vargas et al. (2005) [8]; Ashwood et al. (2010) [25]
	MCP-1/CCL2	AF	↑	Abdallah et al. (2012) [46]
	OPN	Serum	↑	Al-ayadhi and Mostafa (2011) [55]

Tumor necrosis factor	TNF-*α*	Plasma	—	Singh (1996) [39]
		PBMC	↑	Jyonouchi et al. ( 2001) [14] Ashwood et al. (2011) [42, 57]
		CSF	↑	Chez et al. (2007) [4]
		Brain	↑	Li et al. (2009) [26]

Interferon	IFN-*γ*	Plasma	↑	Singh (1996) [39] Croonenberghs et al. (2002) [13]
		Plasma	—	Sweeten et al. (2004) [65]
		Brain	↑	Li et al. (2009) [26]

Growth factors	TGF-*β*1	Plasma	↓	Okada et al. (2007) [72] Ashwood et al. (2008) [67]
	TGF-*β*1	Brain	↑	Vargas et al. (2005) [8]
	EGF	Serum	↓	Suzuki et al. (2007) [78]
	EGF	Serum	↑	Işeri et al. (2011) [79] Tobiasova et al. (2011) [80]
	BDNF	Serum	↑	Nelson et al. (2001) [83]

Growth factors	BDNF	Serum	↑	Miyazaki et al. (2004) [84]
		Serum	↓	Katoh-Semba et al. (2007) [85]
		Brain	↓	Sheikh et al. (2010) [86]
	GM-CSF	Brain	↑	Li et al. (2009) [26]
		PBMC	↑	Ashwood et al. (2008) [67]
		Plasma	↓	Manzardo et al. (2012) [5]

PBMCs: peripheral blood mononuclear cells; AF: amniotic fluid; CSF: cerebrospinal fluid; TGF: transforming growth factor; EGF: epidermal growth factor; BDNF: brain-derived neurotrophic factor; GM-CSF: granulocyte-macrophage colony-stimulating factor.
